# Quantitative dynamic contrast-enhanced parameters and intravoxel incoherent motion facilitate the prediction of TP53 status and risk stratification of early-stage endometrial carcinoma

**DOI:** 10.2478/raon-2023-0023

**Published:** 2023-06-21

**Authors:** Hongxia Wang, Ruifang Yan, Zhong Li, Beiran Wang, Xingxing Jin, Zhenfang Guo, Wangyi Liu, Meng Zhang, Kaiyu Wang, Jinxia Guo, Dongming Han

**Affiliations:** Department of MR, the First Affiliated Hospital of Xinxiang Medical University, Weihui, China; Department of Neurology, the First Affiliated Hospital of Xinxiang Medical University, Weihui, China; MR Research China, GE Healthcare, Beijing, China

**Keywords:** early-stage endometrial carcinoma, dynamic contrast-enhanced MRI, intravoxel incoherent motion, p53 status, risk stratification

## Abstract

**Background:**

The aim of the study was to investigate the value of dynamic contrast-enhanced magnetic resonance imaging (DCE-MRI) and intravoxel incoherent motion (IVIM) in differentiating TP53-mutant from wild type, low-risk from non-low-risk early-stage endometrial carcinoma (EC).

**Patients and methods:**

A total of 74 EC patients underwent pelvic MRI. Parameters volume transfer constant (K^trans^), rate transfer constant (K_ep_), the volume of extravascular extracellular space per unit volume of tissue (V_e_), true diffusion coefficient (D), pseudo-diffusion coefficient (D*), and microvascular volume fraction (f) were compared. The combination of parameters was investigated by logistic regression and evaluated by bootstrap (1000 samples), receiver operating characteristic (ROC) curves, calibration curves, and decision curve analysis (DCA).

**Results:**

In the TP53-mutant group, K^trans^ and K_ep_ were higher and D was lower than in the TP53-wild group; K^trans^, V_e_, f, and D were lower in the non-low-risk group than in the low-risk group (all P < 0.05). In the identification of TP53-mutant and TP53-wild early-stage EC, K^trans^ and D were independent predictors, and the combination of them had an optimal diagnostic efficacy (AUC, 0.867; sensitivity, 92.00%; specificity, 80.95%), which was significantly better than D (Z = 2.169, P = 0.030) and K^trans^ (Z = 2.572, P = 0.010). In the identification of low-risk and non-low-risk early-stage EC, K^trans^, V_e_, and f were independent predictors, and the combination of them had an optimal diagnostic efficacy (AUC, 0.947; sensitivity, 83.33%; specificity, 93.18%), which was significantly better than D (Z = 3.113, P = 0.002), f (Z = 4.317, P < 0.001), K^trans^ (Z = 2.713, P = 0.007), and V_e_ (Z = 3.175, P = 0.002). The calibration curves showed that the above two combinations of independent predictors, both have good consistency, and DCA showed that these combinations were reliable clinical prediction tools.

**Conclusions:**

Both DCE-MRI and IVIM facilitate the prediction of TP53 status and risk stratification in early-stage EC. Compare with each single parameter, the combination of independent predictors provided better predictive power and may serve as a superior imaging marker.

## Introduction

Endometrial carcinoma (EC) is a common malignant tumor of the female reproductive system worldwide, and approximately 80% of newly diagnosed EC patients are in the early stage (International Federation of Gynecology and Obstetrics (FIGO) stage IA, IB).^1^ The TP53 is an important suppressor gene that is deeply involved in tumorigenesis and can control cell growth, apoptosis and regulate angiogenesis. Several studies have shown that high expression of TP53 is closely associated with poor prognosis in EC patients.^2,3^ Risk stratification based on the histologic subtype, grade, FIGO stage, and lymphovascular space invasion (LVSI) is the primary basis for determining treatment strategies for early-stage EC.^4^ For non-low-risk (intermediate-, high-intermediate-, and high-risk) patients, lymphadenectomy (LND) is required in addition to the standard treatment of total hysterectomy with bilateral salpingo-oophorectomy, since it can significantly improve patient benefit. But for low-risk patients, LND is not recommended as it is likely to lead to complications and increased care costs.^5^ Currently, preoperative biopsy and routine magnetic resonance imaging (MRI) are the primary means of obtaining the TP53 status and risk stratification information of EC, respectively.^6^ However, biopsy may not be sufficient for a reliable diagnosis due to shortcomings such as unstable sampling depending on operator experience, inadequate sampling, and invasiveness.^7,8^ At the same time, conventional T1-weighted imaging (T1WI) and T2-weighted imaging (T2WI) not only fail to reflect the TP53 status, histological subtype, and grade information of the lesion but also likely to have a poor to moderate pooled sensitivity in detecting high-risk factors, including deep myometrial invasion and cervical stromal infiltration, due to the presence of adenomyosis and leiomyomas and the loss of the junctional zone.^9,10^ Therefore, finding a noninvasive and effective means to assess the TP53 status and risk stratification in early-stage EC is of great benefit to patients.

Dynamic contrast-enhanced MRI (DCE-MRI) is a promising quantitative MRI sequence that can detect blood supply in biological tissues by analyzing the dynamic distribution of contrast agents through pharmacokinetic models.^11,12^ Intravoxel incoherent motion (IVIM) can also be used to reflect blood perfusion, and compared to DCE-MRI, it not only eliminates the need for contrast agents but also provides additional information on the diffusion of water molecules within the lesion.^13,14,15,16^ Recently, some authors have used IVIM and DCE-MRI for EC-related studies. For example, Satta *et al*. and Fu *et al*. applied IVIM and DCE-MRI to assess the grade, stage, and other histopathological features of EC and showed that some of the derived parameters helped to identify the histopathological features of EC.^17,18^ Zhang *et al*. and Meng *et al*. used IVIM^19,20^, while Ye *et al.*^12^ used DCE-MRI for the preoperative risk assessment of EC, and their results showed that some parameters of DCE-MRI or IVIM could play a positive role in the risk stratification prediction of EC. However, not only did none of these studies address TP53 status but also risk stratification was assessed either by applying only one of the IVIM or DCE-MRI techniques or the subjects were not early-stage EC.

The purpose of this study was to investigate the contributory value of quantitative parameters derived from DCE-MRI and IVIM in differentiating TP53-mutant from TP53-wild, low-risk from non-low-risk early-stage EC, offering a potential reference for the clinical management of early-stage EC.

## Patients and methods

### Study patients

**FIGURE 1.**
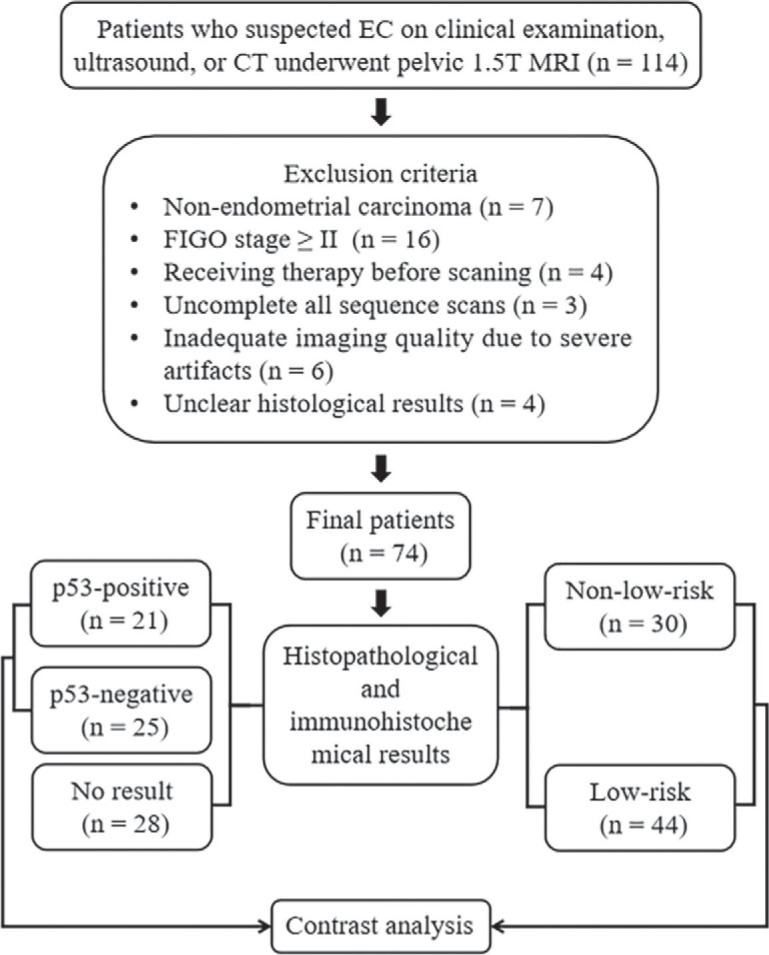
Flowchart of the present study. EC = endometrial carcinoma

This prospective study was complied with ethical committee standards and approved by the ethics committee of the First Affiliated Hospital of Xinxiang Medical University (NO. EC-022-002) and informed consent was taken from all individual participants. From January 2021 to April 2022, 114 female patients underwent pelvic MRI due to suspected EC by clinical examination, ultrasound (US), or computed tomography (CT). Forty participants were excluded during this study: 1) 7 patients were diagnosed with an endometrial polyp, atypical hyperplasia, or other non-EC diseases; 2) 16 patients had FIGO stage ≥ II; 3) 4 patients received radiotherapy or neoadjuvant chemotherapy; 4) 3 patients had claustrophobia or other diseases that prevented them from completing all the sequences; 5) 6 patients had inadequate DCE-MRI or IVIM imaging quality for analysis due to severe artifacts, and 6) 4 patients decided to perform histological analysis and treatment in other institutes. Ultimately, 74 patients were enrolled in the study (Figure 1).

### MRI protocols

A 1.5 T MR system (Optima MR360, Waukesha, WI, USA) with a 12-channel phased-array body coil was used in this study. The imaging protocol included oblique axial (perpendicular to the long axis of the uterus) T1WI, T2WI, DWI, IVIM, and DCE-MRI. For DWI and DCE-MRI sequences, the scans covered the anterior superior iliac spine to the symphysis pubis. For IVIM (b = 0, 20, 40, 80, 160, 200, 400, 600, 800, and 1000 s/mm^2^), to minimize scan time, the scan was limited to the lesion area (determined by an experienced radiologist from the DWI images), and its location, layer thickness, and layer spacing were consistent with the corresponding layer of DWI.^18^ DCE-MRI was performed by a three-dimensional liver acquisition with volume acceleration (3D-LAVA) sequence with 40 phases (time resolution, 9s), and gadopentetate dimeglumine (Gd-DTPA, Bayer Pharmaceutical, Berlin, Germany) was injected intravenously with an automatic injector (0.2 mL/kg, 3.0 mL/s). The protocol details are provided in Table 1.

**TABLE 1.** Imaging protocol parameters

**Parameters**	**T1WI**	**T2WI**	**DWI**	**IVIM**	**DCE-MRI**
Sequence	2D-FSE	2D-FSE	2D-SS-EPI	2D-SS-EPI	3D-LAVA
Orientation	Oblique Axial	Oblique Axial	Oblique Axial	Oblique Axial	Oblique Axial
TR/TE (ms)	659/12.3	6000/95	3708/74.3	2000/80.7	3.5/1.7
FOV (cm^2^)	40 × 40	40 × 40	40 × 40	40 × 40	36 × 36
Matrix	288 × 192	320 × 320	96 × 128	128 × 192	288 × 192
Flip angle (°)	160	160	90	90	15
Slice thickness (mm)	6	6	6	6	6
No. of sections	20	20	20	Based on lesion's size	26
NEX	1	1	1, 4	1, 1, 1, 1, 1, 1, 2, 4, 4, 6	0.73
Fat suppression	/	STIR	STIR	STIR	FLEX
b-values (s/mm^2^)	/	/	0, 800	0, 20, 40, 80, 160, 200, 400, 600, 800, 1000	/
Respiratory compensation	Free	Free	Free	Free	Free
Scan time	1 min 56 s	48 s	1 min 04 s	3~6min	6 min 08 s (40 phases)

DCE-MRI = dynamic contrast-enhanced magnetic resonance imaging; DWI = diffusion-weighted imaging; FOV = field of view; FLEX = FLEXible; FSE = fast spin echo; IVIM = intravoxel incoherent motion; LAVA = liver acquistion with volume assessmeNT; NEX = number of excitations; SS-EPI = single shot echo planar imaging; STIR = short-inversion time(TI) recovery; TR/TE = repetition time/echo time; T1WI = T1-weighted imaging; T2WI = T2-weighted imaging

### Image postprocessing

All images were transferred to the Advantage Workstation (version 4.7), and the IVIM and DCE-MRI images were analyzed within the workstation using vendor-provided software named MADC and GenIQ, respectively. The IVIM parameters were calculated by the following formula:

[1] $${\text{S}}_{\text{b}}/{\text{S}}_{\text{0}}=\left(1-\text{f}\right)\times \text{exp}\left(-\text{b}\times \text{D}\right)+\text{f}\times \text{exp}\left(-\text{b}\times \text{D}*\right)$$

where S_0_ was the signal intensity at the b value of 0; S_b_ was the signal intensity at the b value denoted by the subscript; D was the true diffusion coefficient of a water molecule; D* was the pseudo-diffusion coefficient due to microcirculation; and f was the microvascular volume fraction, indicating the fraction of diffusion related to microcirculation.^11^ The DCE-MRI perfusion parameters were quantitatively calculated based on the Tofts model. The arterial input function (AIF) was obtained from the internal iliac artery. The imaging parameter K^trans^, known as the volume transfer constant, represents the diffusion of contrast medium from the vessel to the extravascular extracellular space (EES); K_ep_, known as the rate transfer constant, represents the diffusion of contrast medium from the EES to the vessel; and V_e_ represents the volume of EES per unit volume of tissue ^13^; thus, K_ep_ = K^trans^/V_e_.

For regions of interest (ROI), first, images of DCE-MRI and IVIM were co-registered, and then on the DCE-MRI images of the phase with the clearest lesion display^21^, ROIs were delineated layer by layer for all slices containing the tumor, and these ROIs were manually drawn along the inside margin of the primary tumor, avoiding areas with cystic degeneration, necrosis, apparent signs and hemorrhage artifacts, and blood vessels. Subsequently, all completed ROIs were automatically copied to the pseudo-color maps of the DCE-MRI and IVIM-derived parameters to calculate the mean values based on the volume of interest (VOI). All of these procedures were completed independently by two radiologists with 7 and 15 years of experience who were blinded to each other's results and the patient's clinicopathological data.

### Histopathologic analysis

All lesion specimens were obtained surgically, and the median interval from pelvic MRI examination to surgery was 12 days (1–25 days). The specimens were processed by an experienced pathologist. The histological subtype, grade, and LVSI were confirmed by hematoxylin/eosin (HE) staining. The stage was estimated with the FIGO staging system.^22^ According to the European Society for Medical Oncology (ESMO) clinical practice guidelines, low-risk patients were classified into the low-risk group, while intermediate-risk, high-intermediate-risk, and high-risk patients were classified into the non-low-risk group.^4^ The TP53 status was evaluated by immunohistochemical (IHC) staining, where non-staining was viewed as the wild group, and faint, moderate, and strong staining was viewed as the mutant group. Ultimately, risk stratification was evaluated in all 74 patients, and TP53 status was evaluated in 46 patients (28 patients declined IHC for financial or other reasons).

### Statistical analysis

All data were analyzed with Stata version 16.0 (Stata Corp) and MedCalc version 15.0 (MedCalc Software). P < 0.05 was considered statistically significant. The interobserver consistency of two radiologists was classified using the intraclass correlation coefficient (ICC) as poor (ICC < 0.40), fair (0.40 ≤ ICC < 0.60), good (0.60 ≤ r < 0.75), or excellent (ICC ≥ 0.75).^23^ The Shapiro–Wilk test was employed to check the normality of the data. The Mann–Whitney U test and the independent samples t-test were used for nonnormally distributed data (median and interquartile range) and normally distributed data (mean ± standard deviation), respectively. The area under the receiver operating characteristic (ROC) curve (AUC) was employed to quantify the diagnostic efficacy of different parameters, and the differences were assessed using DeLong analysis. The combination of parameters was investigated by logistic regression, evaluated by bootstrap (random number set 123, repeated sampling 1000 times, backward strategy, bounded by a value of 0.1), calibration curves, and decision curve analysis (DCA).^24^

## Results

### Basic information

**FIGURE 2.**
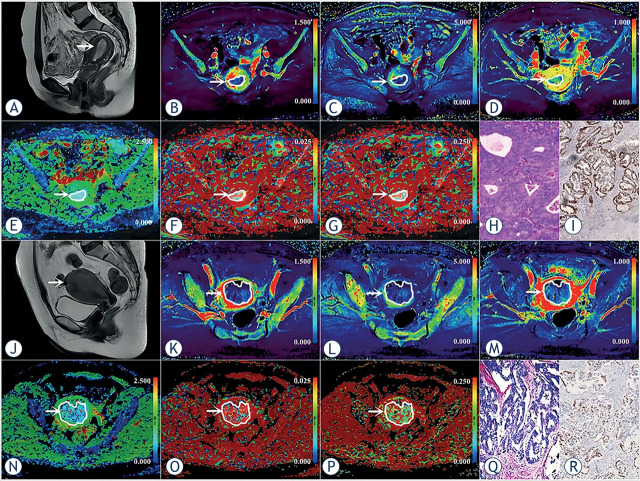
**(A–I)** A 53-year-old woman with low-risk endometrial carcinoma (EC) (arrowheads, endometrioid type, grade 2, stage IA, lymphovascular space invasion (LVSI) negative, and TP53-wild). **(J–R)** A 56-year-old woman with non-low-risk (intermediate) EC (arrowheads, endometrioid type, grade 1, stage IB, LVSI negative, and TP53-mutant). **(A, J)** Sagittal T2-weighted imaging maps; **(B, K)** Oblique axial pseudo colored maps of volume transfer constant (K^trans^); **(C, L)** Oblique axial pseudo colored maps of rate transfer constant (K_ep_); **(D, M)** Oblique axial pseudo colored maps of the volume of extravascular extracellular space per unit volume of tissue (V_e_); **(E, N)** Oblique axial colored maps of true diffusion coefficient (D); **(F, O)** Oblique axial colored maps of pseudo-diffusion coefficient (D^*^); **(G, P)** Oblique axial colored maps of microvascular volume fraction (f), and **(H, Q)** Histopathological images (magnification = 100), and **(I, R)** Immunohistochemical image (magnification = 200).

The clinicopathological and imaging characteristics are shown in Table 2 and Figure 2, respectively.

**TABLE 2.** Clinicopathologic features of the patients

**Variable**	**Data**
Age (mean ± SD) (years)	54.00 ± 7.91
Maximum diameter (mean ± SD) (mm)	25.10 (13.76, 42.58)
FIGO stage n (%)	
IA	44 (59.46)
IB	30 (40.54)
Histologic subtype n (%)	
Adenocarcinoma	67 (90.54)
Non-adenocarcinoma	7 (9.46)
Clear-cell	3 (4.06)
Undifferentiated carcinoma	2 (2.70)
Carcinosarcoma	2 (2.70)
Lymphovascular space invasion n (%)	
Positive	10 (6.76)
Negative	64 (93.24)
Histologic grade n (%)	
Grade 1	54 (72.98)
Grade 2	10 (13.51)
Grade 3	10 (13.51)
Risk stratification n (%)	
Low	44 (59.46)
Intermediate	20 (27.03)
High-intermediate	0 (0.00)
High	10 (13.51)
TP53 expression	
Mutant	21 (28.38)
Wild	25 (33.78)
No result	28 (37.84)

FIGO = International Federation of Gynecology and Obstetrics; SD = standard deviation

### Interobserver consistency

The D, D*, f, K^trans^, V_e_, and K_ep_ measured by 2 radiologists had excellent consistency, and the ICCs were 0.864 (95% CI: 0.788 – 0.913), 0.799 (95% CI: 0.696 – 0.867), 0.855 (95% CI: 0.729 – 0.918), 0.868 (95% CI: 0.799 – 0.915), 0.834 (95% CI: 0.748 – 0.892), and 0.828 (95% CI: 0.739 – 0.888), respectively. The average results were used for the ultimate analysis.

### Differences in parameters

**FIGURE 3.**
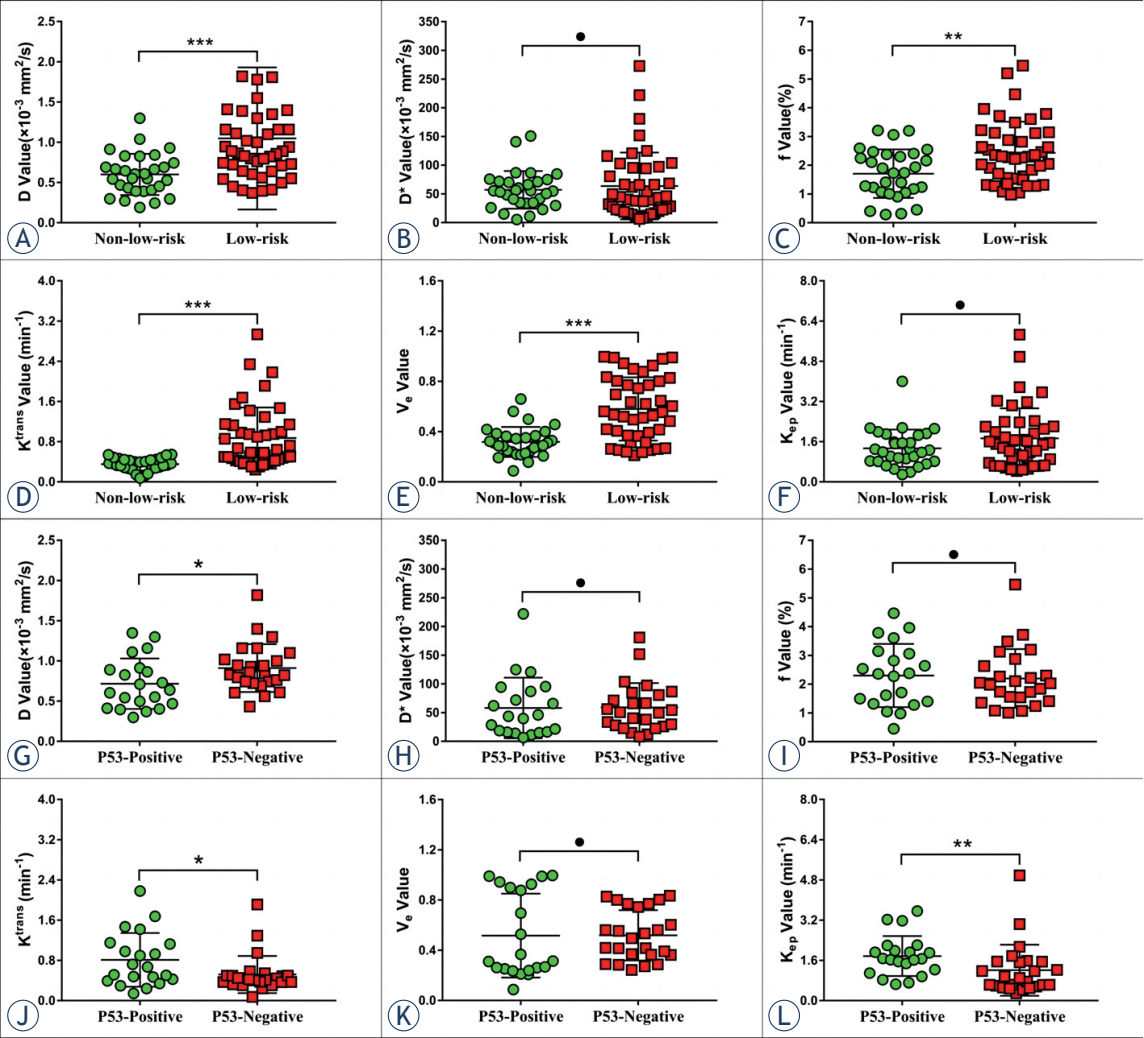
Plots show individual data points, averages, and standard deviations of true diffusion coefficient (D) **(A, G)**, pseudo-diffusion coefficient (D^*^) **(B, H)**, microvascular volume fraction (f) **(C, I)**, volume transfer constant (K^trans^) **(D, J)**, the volume of extravascular extracellular space per unit volume of tissue (V_e_) **(E, K)**, and rate transfer constant (K_ep_) **(F, L)** in low-risk and non-low-risk groups **(A–F)**, TP53-mutant and TP53-wild groups **(G–L)**. Individual points are averages of values calculated by 2 readers. ^*^P < 0.05, ^**^P < 0.01, ^***^P < 0.001, and ^●^ P > 0.005.

The K^trans^ and K_ep_ were higher and D was lower in the TP53-mutant group than in the TP53-wild group (P = 0.038, 0.002, and 0.037, respectively), f, D*, and V_e_ were not significantly different between the two groups (P = 0.750, 0.604, and 0.434, respectively). The K^trans^, V_e_, f, and D values were lower in the non-low-risk group than in the low-risk group (P < 0.001, < 0.001, 0.002, and < 0.001, respectively), K_ep_ and D* were not significantly different between the two groups (P = 0.218 and 0.601) (Table 3, Figure 3).

**TABLE 3.** Comparison of different parameters

**Parameters**	**D (×10^−3^mm^2^/s)**	D^*^ (×10^−3^mm^2^/s)	**f (%)**	**K^trans^ (min^−1^)**	**V_e_**	**K_ep_(min^−1^)**
Risk stratification						
High-risk (n = 10)	0.63 (0.40, 0.73)	58.40 (40.10, 88.73)	1.64 ± 0.60	0.35 (0.15, 0.43)	0.30 ± 0.07	1.23 (0.48, 1.54)
High-intermediate-risk (n = 0)	/	/	/	/	/	/
Intermediate-risk (n = 20)	0.55 (0.40, 0.81)	52.00 (26.88, 74.33)	1.74 ± 0.96	0.37 (0.29, 0.47)	0.33 ± 0.14	1.24 (0.82, 1.98)
Low-risk (n = 44)	0.86 (0.64, 1.16)	44.35 (21.93, 95.33)	2.43 ± 1.08	0.61 (0.43, 1.14)	0.58 ± 0.25	1.53 (0.79, 2.21)
P-value	**0.033** ^a^	0.464 ^a^	**0.012** ^a^	**< 0.001** ^a^	**< 0.001** ^a^	0.191 ^a^
P-value (High *vs* Intermediate)	0.880 ^b^	0.248 ^b^	0.735 ^c^	0.397 ^b^	0.532 ^c^	0.307 ^b^
P-value (High *vs* Low)	**0.009** ^b^	0.238 ^b^	**0.004** ^c^	**< 0.001** ^b^	**0.001** ^c^	0.099 ^b^
P-value (Intermediate vs Low)	**0.001** ^b^	0.937 ^b^	**0.014** ^c^	**< 0.001** ^b^	**< 0.001** ^c^	0.582
Low-risk (n = 44)	0.86 (0.64, 1.16)	44.35 (21.93, 95.33)	2.43 ± 1.08	0.61 (0.43, 1.14)	0.58 ± 0.25	1.53 (0.79, 2.21)
Non-low-risk (High + Intermediate, n = 30)	0.58 (0.40, 0.77)	55.25 (34.63, 72.78)	1.71 ± 0.84	0.37 (0.28, 0.45)	0.32 ± 0.12	1.23 (0.82, 1.87)
z/t value	− 3.793	−0.523	3.234	−5.109	5.304	−1.233
P-value	**< 0.001** ^b^	0.601 ^b^	**0.002** ^c^	**< 0.001** ^b^	**< 0.001** ^c^	0.218 ^b^
TP53 expression						
Mutant (n = 21)	0.72 ± 0.31	43.70 (16.30, 90.75)	2.30 ± 1.09	0.67 (0.41, 1.14)	0.32 (0.25, 0.91)	1.67 (1.17, 2.09)
Wild (n = 25)	0.91 ± 0.29	50.60 (26.90, 82.75)	2.20 ± 1.02	0.43 (0.37, 0.49)	0.49 (0.36, 0.76)	0.90 (0.58, 1.55)
Z/t value	−2.155	−0.518	0.321	−2.073	−0.783	−3.165
P-value	**0.037** ^c^	0.604 ^b^	0.750 ^c^	**0.038** ^b^	0.434 ^b^	**0.002** ^b^

The bold typeface in the table indicates the comparison with statistical significance.

a Comparisons were performed by *Analysis* of variance (ANOVA) test;

b comparisons were performed by Mann–Whitney U test;

c comparisons were performed by independent t test.

### Regression analyses

In the identification of TP53-mutant and TP53-wild early-stage EC, the potential related factors such as age, tumor size, risk stratification, FIGO stage, subtype, grade, LVSI, D, D*, f, K^trans^, V_e_, and K_ep_ were all enrolled in regression analysis. Univariate analysis demonstrated that grade, D, K^trans^, and K_ep_ were all risk predictors (P all < 0.1), while multivariate analysis showed that only D and K^trans^ were independent predictors (P = 0.003, 0.016).

In the identification of non-low-risk and low-risk early-stage EC, potential risk-related factors such as age, tumor size, TP53 status, D, D*, f, K^trans^, V_e_, and K_ep_ were all enrolled in regression analysis. Univariate analysis demonstrated that tumor size, D, f, K^trans^, and V_e_ were all risk predictors (P all < 0.1), while multivariate analysis showed that only f, K^trans^, and V_e_ were independent predictors (P = 0.036, 0.003, and 0.024, respectively) (Table 4).

**TABLE 4.** Logistic regression analyses

**Parameters**	**Univariate Analyses**	***P*-value**	**Multivariate Analyses**	***P*-value**
	
	**OR for 1 SD (95% CI)**		**OR for 1 SD (95% CI)**	
Low vs non-low risk				
Age (year)	1.462 (0.894–2.388)	0.130	/	/
Tumor size (mm)	1.055 (1.003–1.110)	**0.038**	1.083 (0.979–1.197)	0.123
TP53 mutant	1.506 (0.407–5.578)	0.540	/	/
D (×10^−3^mm^2^/s)	0.089 (0.021–0.373)	**0.001**	0.144 (0.015–1.334)	0.088
D^*^ (×10^−3^mm^2^/s)	0.867 (0.533–1.412)	0.567	/	/
f (%)	0.419 (0.226–0.776)	**0.006**	0.292 (0.093–0.921)	**0.036**
K^trans^ (min^−1^)	0.009 (0.001–0.153)	**0.001**	0.001 (0.000–0.089)	**0.003**
V_e_	0.173 (0.069–0.432)	**< 0.001**	0.130 (0.022–0.766)	**0.024**
K_ep_ (min^−1^)	0.642 (0.367–1.126)	0.122	/	/
TP53 mutant vs wild				
Age (year)	0.855 (0.465–1.548)	0.605	/	/
Tumor size (mm)	1.175 (0.649–2.127)	0.594	/	/
Subtype	77.708 (0.001–100.5)	0.999	/	/
Grade	2.099 (0.957–4.602)	**0.064**	1.961 (0.816–4.717)	0.132
Risk stratification	1.506 (0.407–5.578)	0.540	/	/
FIGO stage	1.360 (0.739–2.505)	0.323	/	/
LVSI	802.578 (0.001–1150.5)	0.999	/	/
D (×10^−3^mm^2^/s)	2.063 (1.016–4.191)	**0.045**	8.274 (2.066–33.136)	**0.003**
D^*^ (×10^−3^mm^2^/s)	1.020 (0.567–1.835)	0.948	/	/
f (%)	0.906 (0.504–1.629)	0.742	/	/
K^trans^ (min^−1^)	0.487 (0.236–1.003)	**0.051**	0.155 (0.034–0.710)	**0.016**
V_e_	1.008 (0.560–1.812)	0.979	/	/
K_ep_ (min^−1^)	0.501 (0.244–1.032)	**0.061**	1.172 (0.425–3.234)	0.759

D = true diffusion coefficient; D^*^ = pseudo-diffusion coefficient; f = microvascular volume fraction; FIGO = international federation of gynecology and obstetrics; CI = confidence interval; K_ep_ = rate transfer constant; K^trans^ = volume transfer constant; LVSI = lymphovascular space invasion; OR = odds ratio;. SD = standard deviation; V_e_ = volume of extravascular extracellular space per unit volume of tissue

The bold typeface in the table indicates the logistic regression analyses with statistical significance.

In the analysis of the high- and low-risk group, the TP53 mutant data were analysed only for these patients who had the p53 gene test. The remaining parameters, such as diameter, were analysed for all 74 patients.

### Diagnostic performance of different parameters

**FIGURE 4.**
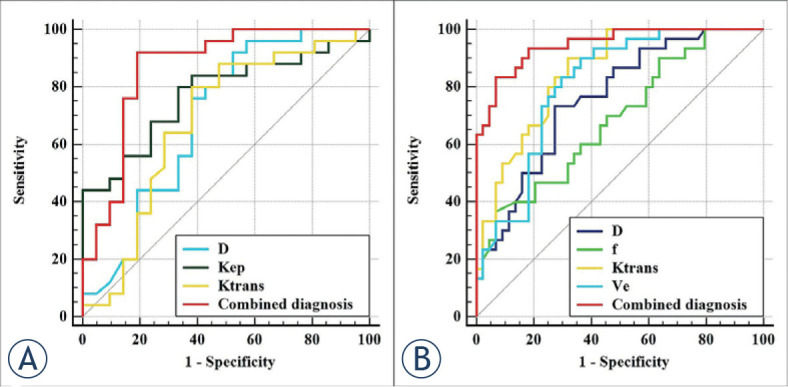
Receiver operating characteristic (ROC) curves, **(A)** shows each parameter and the combination of independent predictors for differentiation of TP53-mutant and TP53-wild early-stage endometrial carcinoma (EC); **(B)** shows each parameter and the combination of independent predictors for differentiation of low-risk and non-low-risk early-stage EC.

In the differentiation of TP53-mutant and TP53-wild early-stage EC, the combination of independent predictors (K^trans^ and D) showed the optimal diagnostic efficacy (AUC = 0.867; sensitivity, 92.00%; specificity, 80.95%; P < 0.001), which was significantly better than D (AUC = 0.694, Z = 2.169, P = 0.030), and K^trans^ (AUC = 0.679, Z = 2.572, P = 0.010). However, the difference between the combination of independent predictors and K_ep_ (AUC = 0.773) was not significant (AUC = 0.773, Z = 1.272, P = 0.203) (Figure 4A, Table 5).

**TABLE 5.** Predictive performance of different parameters

**Parameters**	**AUC (95% CI)**	**P-value**	**Cutoff**	**Sensitivity**	**Specificity**	**Comparison with combined diagnosis**
Low vs non-low risk						
D (×10^−3^mm^2^/s)	0.761 (0.648–0.853)	< 0.001	0.691	73.33%	72.73%	Z = 3.113, P = 0.002
D^*^ (×10^−3^mm^2^/s)	0.536 (0.416–0.653)	0.598	/	/	/	/
f (%)	0.688 (0.569–0.790)	0.003	1.240	36.67%	93.18%	Z = 4.317, P < 0.001
K^trans^ (min^−1^)	0.852 (0.750–0.924)	< 0.001	0.487	90.00%	68.18%	Z = 2.713, P = 0.007
V_e_	0.808 (0.700–0.890)	< 0.001	0.401	83.33%	70.45%	Z = 3.175, P = 0.002
K_ep_ (min^−1^)	0.585 (0.652–0.849)	0.204	/	/	/	/
Combined diagnosis 1	0.947 (0.869–0.986)	< 0.001	/	83.33%	93.18%	/
TP53 mutant vs wild						
D (×10^−3^mm^2^/s)	0.694 (0.541–0.821)	0.019	0.605	92.00%	47.62%	Z = 2.169, P = 0.030
D^*^ (×10^−3^mm^2^/s)	0.545 (0.391–0.692)	0.498	/	/	/	/
f (%)	0.535 (0.382–0.648)	0.388	/	/	/	/
K^trans^ (min^−1^)	0.679 (0.525–0.809)	0.036	0.499	80.00%	61.90%	Z = 2.572, P = 0.010
V_e_	0.568 (0.413–0.713)	0.675	/	/	/	/
K_ep_ (min^−1^)	0.773 (0.626–0.884)	< 0.001	1.557	80.00%	66.67%	Z = 1.272, P = 0.203
Combined diagnosis 2	0.867 (0.734–0.949)	< 0.001	/	92.00%	80.95%	/

AUC = *area* under the receiver operating characteristic (ROC) curve; D = true diffusion coefficient; D^*^ = pseudo-diffusion coefficient; f = microvascular volume fraction; K_ep_ = rate transfer constant; K^trans^ = volume transfer constant; V_e_ = volume of extravascular extracellular space per unit volume of tissue

The combined diagnosis 1 represents f + K^trans^ + V_e_; the combined diagnosis 2 represents D + V_e_

In the differentiation of low-risk and non-low-risk early-stage EC, the combination of independent predictors (f, K^trans^, and V_e_) showed the optimal diagnostic efficacy (AUC = 0.947; sensitivity, 83.33%; specificity, 93.18%; P < 0.001), which was significantly better than D (AUC = 0.761, Z = 3.113, P = 0.002), f (AUC = 0.688, Z = 4.317, P < 0.001), K^trans^ (AUC = 0.852, Z = 2.713, P = 0.007), and V_e_ (AUC = 0.808, Z = 3.175, P = 0.002) (Figure 4B, Table 5).

### Validation

**FIGURE 5.**
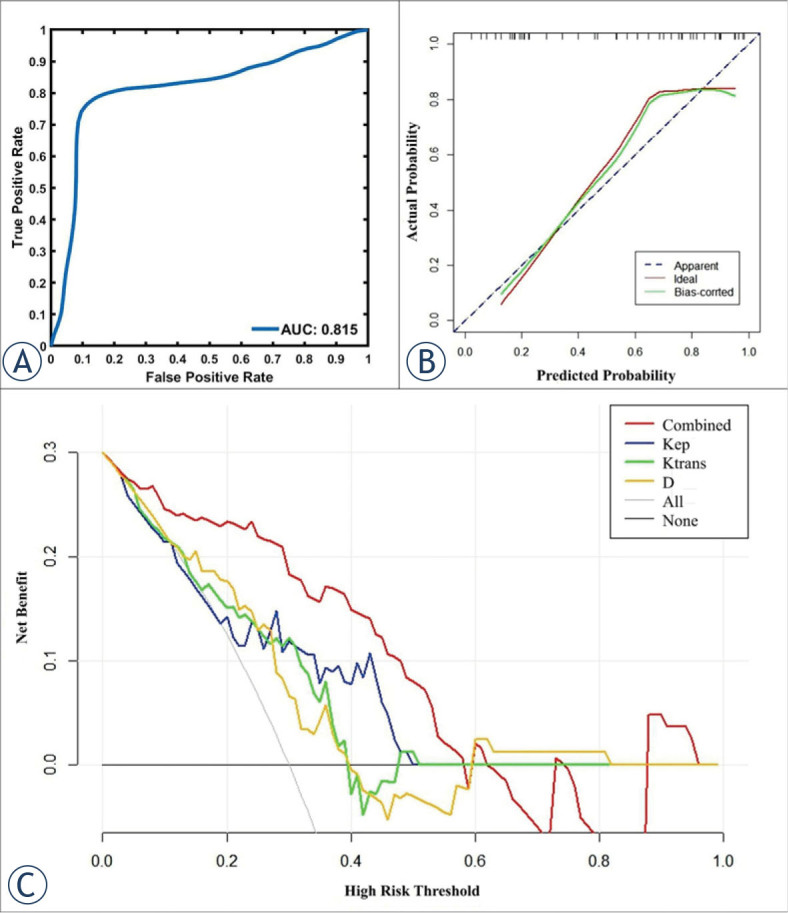
In the prediction of TP53 status, receiver operating characteristic curves **(A)**, calibration curves **(B)**, and decision curve analysis **(C)** of the validation model.

**FIGURE 6.**
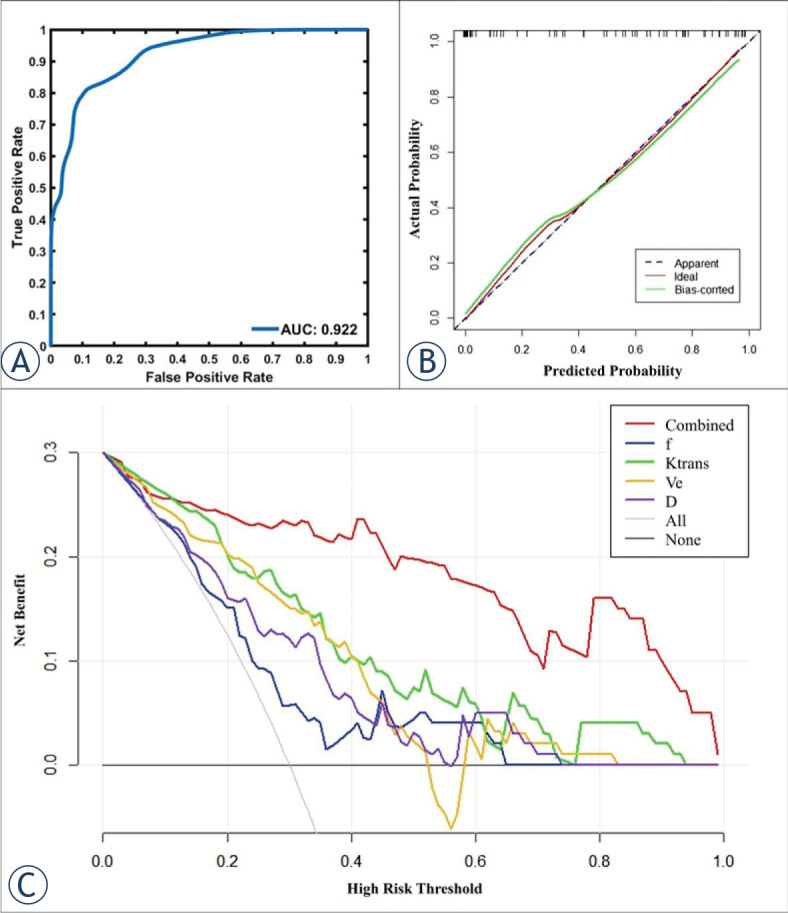
In the prediction of risk stratification, receiver operating characteristic curves **(A)**, calibration curves **(B)**, and decision curve analysis **(C)** of the validation model.

Bootstrapped samples were used to validate the combination of independent predictors. The ROC and the calibration curve indicated that the validation models not only had high accuracy in identifying TP53-mutant and TP53-wild early-stage EC (AUC, 0.815; 95% CI, 0.782 – 0.846, Figure 5A), and low-risk and risk early-stage EC (AUC, 0.922; 95% CI, 0.895 – 0.940, Figure 6A), but also highly had good consistency (Figure 5B, 6B). Also, DCA showed that the above combinations of independent predictors were both reliable clinical decision tools (Figure 5C, Figure 6C).

## Discussion

### Prediction of TP53 status and risk stratification of early-stage EC by IVIM

The parameter D of IVIM can reflect the diffusion movement of water molecules in the tissue, and usually, the more obvious the restriction of water molecule diffusion, the smaller the D value.^13^ In this study, the D value of the TP53-mutant group was significantly lower than that of the TP53-wild group, which was similar to the results of Wang *et al*. in the field of epithelial ovarian cancer^25^, suggesting that D values can be used to predict TP53 status of early-stage EC. Presumably, the reason was that TP53-mutant has a faster rate of cell proliferation than TP53-wild, which easily impedes the diffusion of water molecules, resulting in a lower D value.^26^ In addition, D could also be used to assess the risk stratification of early-stage EC in the present study, which was consistent with previous studies.^17,19^ The reason may be that there were differences in histological grade, FIGO stage, and lymph node metastasis between the low-risk and non-low-risk early-stage EC, resulting in different degrees of influence on the diffusion of water molecules and ultimately leading to significant differences in D values between the two groups.^18,20^

D* was a perfusion parameter of IVIM that is mainly correlated with the velocity of blood flow within the microcirculation.^13^ Previous publications have demonstrated that D* values with poor stability and repeatability could not effectively evaluate histopathological information of early-stage EC due to the influence of the scanning parameters, the ROI determination method, the signal-to-noise ratio (SNR), and other factors.^17,18,19,20^ In this study, there was no statistically significant difference in D* between the TP53-mutant and TP53-wild groups, and the low-risk and the non-low-risk groups, which was consistent with the above research, further proving that the D* value was unable to play a role in the assessment of TP53 status and risk stratification in early-stage EC.

As another perfusion parameter derived from IVIM, f was mainly related to the microvascular density of the tissue.^13,27^ A study by Zhang *et al*. involving 53 participants showed that although high-risk early-stage EC is metabolically active and rich in neovascularization, due to the dense tissue structure and more necrotic tissue, its overall internal microvascular density is instead reduced compared to that of low-risk early-stage EC, so the f value decreases.^20^ This trial was conducted on a larger sample size of patients (n = 74) and obtained results consistent with those of Zhang *et al*.^19^ Further analysis also identified the f value as an independent predictor for discriminating between low-risk and non-low-risk early-stage EC. However, there were also studies that have shown conflicting results of f values in the assessment of lesions. For example, the study by Meng *et al*. showed that high-risk early-stage EC had higher f values than low-risk early-stage EC.^20^ Similarly, in the assessment of gliomas, the study of Bai *et al*. showed that low-grade gliomas had higher f values than high-grade gliomas^14^, while Shen *et al*. concluded that high-grade gliomas have greater f values.^16^ We speculate that the above phenomenon may be caused by the variations in scanning equipment and b-value settings^28^, as well as the shortcoming that the f value itself is susceptible to T2 contribution and relaxation effects.^29^ In addition, the results of this study also showed that the f was similar to D* and could not differentiate TP53-mutant from TP53-wild early-stage EC, which to some extent suggests that the use of IVIM perfusion parameters to assess the TP53 status of early-stage EC may still need further exploration.

### Prediction of TP53 status and risk stratification of early-stage EC by DCE-MRI

K^trans^ is the most significant perfusion-related parameter in DCE-MRI, mainly reflecting the transfer rate of the contrast agent from the vessel to the EES.^30^ Previous studies have shown that the more neovascularization in the tissue and the greater the permeability, the greater the K^trans^ value.31 In terms of TP53 status assessment, the present study found a significantly higher K^trans^ value in the TP53-mutant group compared with the TP53-wild group, which we suggest may be related to the ability of TP53 gene overexpression to promote angiogenesis.^2,3^ In terms of risk stratification assessment, several studies have shown that EC with aggressive characteristics, such as grade 3, advanced FIGO stage, and non-endometrioid sub-type, grows quickly without sufficient neoangiogenesis (i.e., blood support), resulting in tissue hypoxia. Hypoxia will lead to tissue necrosis and the formation of hypoperfused areas, thus eventually causing a decrease in overall tumor perfusion and a decrease in K^trans^ values.12,17,32,33 In this work, the K^trans^ value was significantly lower in the non-low-risk group than in the low-risk group, which was consistent with the above findings and further demonstrates that the K^trans^ value can play a role in the risk stratification of early-stage EC.

K_ep_ was designed to reflect the transfer rate of the contrast agent from the EES into vessels, so similar to K^trans^, its size was closely related to the number of new vessels and vascular permeability.^29^ In this study, since TP53 overexpression can promote angiogenesis^2,3^, the K_ep_ value of the TP53-mutant group was significantly higher than those of the TP53-wild group, and the diagnostic efficacy was 0.773. However, the K_ep_ value did not show significant value in the identification of different risk stratifications, which was not consistent with the study of Ye *et al.*^12^ We speculated that this may be because the study by Ye *et al*. included both early-stage (stage I) and advanced-stage (stage II, III, and IV) EC, whereas the present study population included only early-stage EC, which reduced the differences in patients between the different groups and ultimately resulted in nonfunctional K_ep_ values.

V_e_ is a parameter in DCE-MRI that can reflect the volume of EES. In the present study, there was no significant difference in V_e_ between the TP53-mutant and TP53-wild groups, which may be related to the fact that TP53 overexpression promotes both cell proliferation and angiogenesis, resulting in difficulty in significant changes in EES.^2,3^ In terms of risk stratification assessment, V_e_ values in the non-low-risk group were significantly smaller than those in the low-risk group, which was similar to the results of previous studies^17,34^, and we speculated that the reason for this result may lie in the fact that the non-low-risk group had greater invasiveness and therefore greater cell density, tighter tissue structure, and smaller EEC compared with the low-risk group. However, some studies have also concluded that V_e_ was difficult to use in the evaluation of diseases such as EC and breast cancer.^12,35^ This may be related to the fact that Ve is less stable and susceptible to factors such as lesion edema and microcystic changes.^36^ In a follow-up study, we will expand the sample size and further explore the role of V_e_ in EC assessment to obtain more convincing results.

### Diagnostic performance comparison

The diagnostic efficacy of the combination of independent predictors and each individual parameter was compared in this study, and the results showed that the diagnostic efficacy of the former was significantly higher than that of the latter, which may be because the combination of independent predictors concentrates the advantages of different parameters and therefore can reflect the lesion characteristics more comprehensively and accurately. Therefore, we suggest that the combined application of IVIM and DCE-MRI in clinical routine may provide a more reliable basis for the TP53 status and risk stratification prediction of early-stage EC when conditions permit.

### Correlation of risk stratification with TP53 mutation

In this study, TP53 mutation and risk stratification in early-stage EC were included in each other's regression analysis, and the results showed that neither was a predictor of the other. Although the small sample size may have affected the reliability of the above results to a certain extent, it indicates to some extent that the TP53 status in early-stage EC is not significantly correlated with risk stratification. In the future, as the sample size increases, we will conduct more in-depth studies on the relationship between the two, with a view to obtaining more accurate results.

This study has several limitations. First, our study was designed at a single institution with a relatively small number of patients, especially since some patients forgo immunohistochemical testing for financial reasons, which may have led to selection bias. Second, due to the small sample size, this study did not set up a separate validation set but used the bootstrap (1000 samples) method to validate the combination of independent predictors, which may have reduced the reliability of the experimental results. Third, areas of cystic degeneration, necrosis, apparent signs and hemorrhage artifacts, or vessels were avoided in the delineation of the ROI, which may influence the determination of some parameters. Finally, the machine used in this study was a 1.5 T MRI, and its imaging quality and parameter reliability may be inferior to those of a 3.0 T MRI.

## Conclusions

Both DCE-MRI and IVIM facilitate the prediction of TP53 status and risk stratification in early-stage EC. Comparison with each single parameter, the combination of independent predictors provided better predictive power and may serve as a superior imaging marker.
